# Cryptorchidism in free-living jaguar (*Panthera onca*): first case report

**DOI:** 10.1590/1984-3143-AR2020-0555

**Published:** 2020-11-24

**Authors:** Pedro Nacib Jorge-Neto, Maitê Cardoso Coelho da Silva, Antonio Carlos Csermak-Júnior, Jorge Aparecido Salmão-Júnior, Gediendson Ribeiro de Araújo, Gustavo de Oliveira, Lucas Leuzinger, Cristiane Schilbach Pizzutto, Thyara de Deco-Souza

**Affiliations:** 1 Instituto Reprocon, Campo Grande, MS, Brasil; 2 Faculdade de Medicina Veterinária e Zootecnia, Universidade de São Paulo, São Paulo, SP, Brasil; 3 Faculdade de Medicina Veterinária e Zootecnia, Universidade Federal de Mato Grosso do Sul, Campo Grande, MS, Brasil; 4 Instituto de Biociências, Universidade Federal de Mato Grosso do Sul, Campo Grande, MS, Brasil; 5 Instituto Onças do Rio Negro, Aquidauana, MS, Brasil

**Keywords:** medetomidine, monorchidism, semen quality, testis

## Abstract

Cryptorchidism is a genital alteration wherein one or both testicles fail to descend into the scrotum and has multifactorial causes. A free-range adult male was captured twice in the Pantanal of Nhecolândia to put a GPS collar and semen collection. Pharmacological semen collection, andrological examination and semen analysis were performed. At the first capture and during the andrological examination only the left testis was found, and the male qualified as cryptorchid. The penis had no penile spines at either procedure. The semen volume obtained at first and second capture was 435 and 160 μL, respectively, with a concentration of 618 and 100 x 10^6^ sperm/mL, progressive motility of ~ 5% and ~ 1% and total morphological sperm abnormalities of 74% and 86%. The male was monitored by a GPS collar, but the signal was lost, making it difficult to re-captures and perform new seminal and ultrasound evaluations to discard monorchidism – exceedingly rare in felids. Genetic studies to assess the individual's homozygosity are necessary to verify whether cryptorchidism in this individual has a genetic factor.

## Introduction

Cryptorchidism is a genital alteration wherein one or both testicles fail to descend into the scrotum and has multifactorial causes such as genetic, epigenetic and environmental components (Amann and Veeramachaneni, 2006). Although uncertain if cryptorchidism is linked to other congenital defects in cats (Little, 2011), it has been reported as a genetic problem caused by inbreeding (Mansfield and Land, 2002). However, other causes may lead to cryptorchidism in mammals, such as navel infection during testicular descent (Romagnoli, 1991), exposure of the fetus to increased maternal estrogen (Depue et al., 1983), nonsteroidal antiandrogen drug (Hutson et al., 1994) or even maternal vitamin A deficiency during fetal development (Wilson et al., 1953).

In wild felines, cryptorchidism has been reported in lion (Cohrs and Schneider, 1936), cougar (Mansfield and Land, 2002), eurasian lynx (Ryser-Degiorgis et al., 2004), cheetah (Crosier et al., 2006), fishing cat (Pinyopummin et al., 2011), ocelot (Araujo et al., 2013), black-footed cat (Sliwa et al., 2016) and amur leopard (Napier et al., 2018).

## Methods

An adult free-range male jaguar (*Panthera onca*), estimated age of 4 to 5 years old, was captured in the Pantanal of Nhecolândia (Aquidauana, MS, Brazil; 19°34'45.6”S 56°09'09.5”W) to put a GPS collar for Onças do Rio Negro’s monitoring project and semen collection. It had authorization for scientific activities issued by SISBIO/ICMBio/MMA (no. 57293-5).

The male was captured twice with the foot snare trap technique (Araujo et al., 2020 forthcoming) on November 17, 2019 at ~10:15 PM and March 24, 2020 at ~9:50 AM. Chemical restraint was performed using anesthetic darts fired with a blowpipe and containing ketamine (5 mg/kg; im) and medetomidine (0.1 mg/kg; im) (Araujo et al., 2018). After all the procedures, anesthesia was reversed using yohimbine (0.4 mg/kg; im).

During the andrological examination, testis location, consistency and biometry, as well as penile spines characteristics, were evaluated. The presence of penile spines was subjectively evaluated and rated on a scale of 0 (no penile spines) to 3 (prominently visible) (Araujo et al., 2013).

The testicular volume was measured using a caliper and applying the equation for an ellipsoid 4/3 × π × L/2 × B_1_/2 × B_2_/2, where L, B_1_ and B_2_ are the length and two breadths of the ellipsoid (Harcourt et al., 1995). Skin thickness was also measured and discounted from testicular dimensions. The testicular volume was directly converted to grams since the volumetric density of the mammalian testis is 1.046 (Johnson et al., 1981). Relative testes weight (RTW) was obtained by dividing total testis weight by body weight.

Semen collection was performed by urethral catheterization as described by Araujo et al. (2018). Briefly, 10 to 20 min after medetomidine injection, a disposable semi-rigid tomcat urinary catheter (w/ open end, 3FR, 130 mm long) was introduced carefully into the urethra. A 1-mL syringe was connected to the catheter, and negative pressure was applied to increase the suction effect.

Semen samples were diluted with OptiXcell (IMV Technologies) and a 7,5 µL drop was placed over a sample chip (Aidmics Biotechnology). Then, the samples were subjectively evaluated for motility and vigor (wave motion scoring system, from 0 to 5; Evans and Maxwell, 1987) using an iSperm Semen Analyzer (Aidmics Biotechnology) as a portable microscope. An aliquot was prepared with formol-saline for later sperm concentration – measured in a Neubauer chamber – and morphologic evaluation.

Sperm morphology was evaluated using differential interference phase contrast (DIC) microscopy of wet-mount semen (magnification ×1,250) and at least 200 spermatozoa per ejaculate were examined in random fields. Sperm defects were categorized as either minor or major and their sum as total defects, according to Blom's classification (Blom, 1973).

## Results

**Figure 1:**
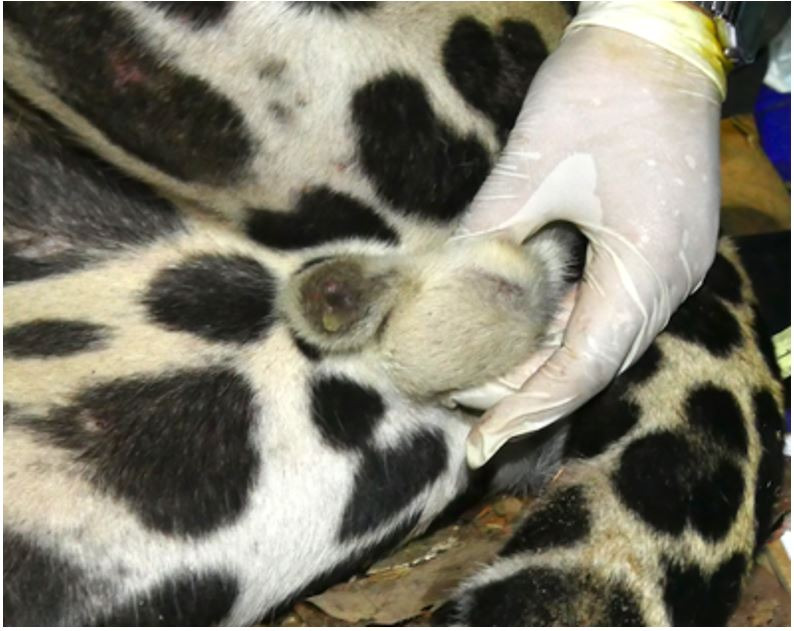
Left testis visible and right testis absent.

**Figure 2:**
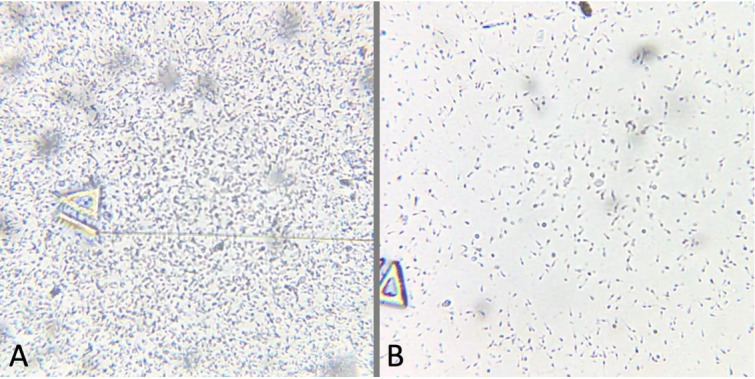
Image of raw semen (without dilution) obtained through the iSperm of the first (A) and second (B) captures.

The jaguar weighed 109 kg and 119 kg at the first and second capture, respectively. During the andrological examination only the left testis was found (Figure 1) presenting a firm consistency in both captures. The left testis measured 3.92 x 2.6 x 2.46 cm (length x width x thickness) with double skinfold thickness of 0.77 cm, resulting in a testicular volume of 13.12 mL or 13.72 g. The right testicle could not be palpated as it was found absent from the scrotum. The penis measured 2.97 x 1.94 cm (length x diameter) and had no penile spines. Sperm volume, motility, vigor, concentration and defects in both captures are reported in Table 1 and iSperm image in Figure 2.

**Table 1:** Seminal parameters from the cryptorchid jaguar male.

**Variable**	**1^st^ Capture**	**2^nd^ Capture**
Volume	435 μL	160 μL
Motility	~ 5%	~ 1%
Vigor (0-5)	1	1
Concentration	618 x 10^6^ sperm/mL	100 x 10^6^ sperm/mL
Total Cells	269 x 10^6^ sperm	16 x 10^6^ sperm
Normal Sperm	26%	14%
Minor Defects	11%	14%
Major Defects	63%	72%
Total Defects	74%	86%
Testis Consistency	firm	firm

## Discussion

To the best or our knowledge, the present study is the first to report cryptorchidism in jaguars. There are still many questions regarding the reproductive biology of jaguar, and the lack of literature is incompatible with its relevance in South and Central America. For example, ovulation forms (Jorge-Neto et al., 2020), puberty and oestral cycle (Viau et al., 2020) and sperm ﻿morphology, morphometry, ultrastructure and mitochondrial activity (Silva et al., 2019) were only reported in the last three years, all in captive animals.

Cryptorchidism is a reproductive disorder defined as the absence of one or both testes inside the scrotum that may be in the abdominal cavity or inguinal area. Monorchidism, defined by the total absence of one testis, is rare in domestic cats and has never been reported in wild felids. For this reason, felids that present with only one scrotal testicle should be considered cryptorchid until proven otherwise (Griffin et al., 2020). Cryptorchidism can have a genetic character caused by inbreeding and can be transmitted to offspring. In Florida panthers, cryptorchid males can have good seminal quality and achieve the same reproductive success as normal males (Mansfield and Land, 2002).

The volume of the left testis of the animal reported was within the average known size (13.72 g), compatible with previously reported in jaguars (13.8 g ± 5.9; Azevedo et al., 2006). Although the semen recovered volume is consistent with that reported by Araujo et al. (2018) in free-living jaguars, the sperm concentration and amount of normal sperm were lower (618 vs. 3,315 x 10^6^ sperm / mL ± 1,174 and 26% and 14% vs. 51% ± 22.8, respectively). Although poor semen quality is not necessarily related to cryptorchid animals, it was found in both captures.

At least three hours before the second capture (~ 9:50 AM), the male had closed proximity interactions with a monitored female, as shown by GPS telemetry. This interaction lasted for 4 days (between March 24 at 6 AM and March 28 at 10 PM). Since jaguar is a multiple copulation species (Jorge-Neto et al., 2018), presumably the low sperm concentration found was due to copulations recently occurring. Therefore, it is unlikely that the poor motility and large number of morphologically abnormal spermatozoa were the results of sexual abstinence. The lack of sperm quality in sexually active males was recently reported in meerkats (Silvatti et al., 2020).

The female with whom the male had physical interactions for 4 days was possibly in estrus. However, the GPS collar stopped working on June 27 – between 92 and 95 days after presumed copulation. Telemetry can indicate pregnant females 90 days after mating (Fontes et al., 2021 forthcoming), but in the last 5 days before the loss of signal the female did not show behavior compatible with pregnancy. Therefore, it is hypothesized that physical interaction may not have resulted in pregnancy. As free-living jaguars’ copulatory behavior – multiple male matting – remains controversial (Cavalcanti and Gese, 2009; Pinho et al., 2014; Soares et al., 2006), it is not possible, at this moment, to predict the impact that a cryptorchid male with low sperm viability can cause to the local population of the species.

The animal in this short report was being monitored by a GPS collar. Since May 18, 2020 the GPS collar has ceased data transmission. However, he was photographed with another female by camera trap on August 07^th^, 2020. Further captures may occur, but for now it is improbable to evaluate seminal quality and ultrasound evaluation to definitively discard monorchidism. Therefore, genetic analysis of this male compared to other jaguars in the same zone for the analysis of eventual inbreeding should be performed.

## Conclusion

We report here the first case of cryptorchidism in a jaguar, in a twice captured free-living animal, with poor seminal parameters at both times.
